# Supplementary figures and images for: The Fate of the Chlorophyll Derivatives in Olives Preserved and/or Packaged in Presence of Exogenous Copper
Source: Molecules. 2023 May 22;28(10):4250. doi: 10.3390/molecules28104250 (PMC10220830; doi:10.3390/molecules28104250)

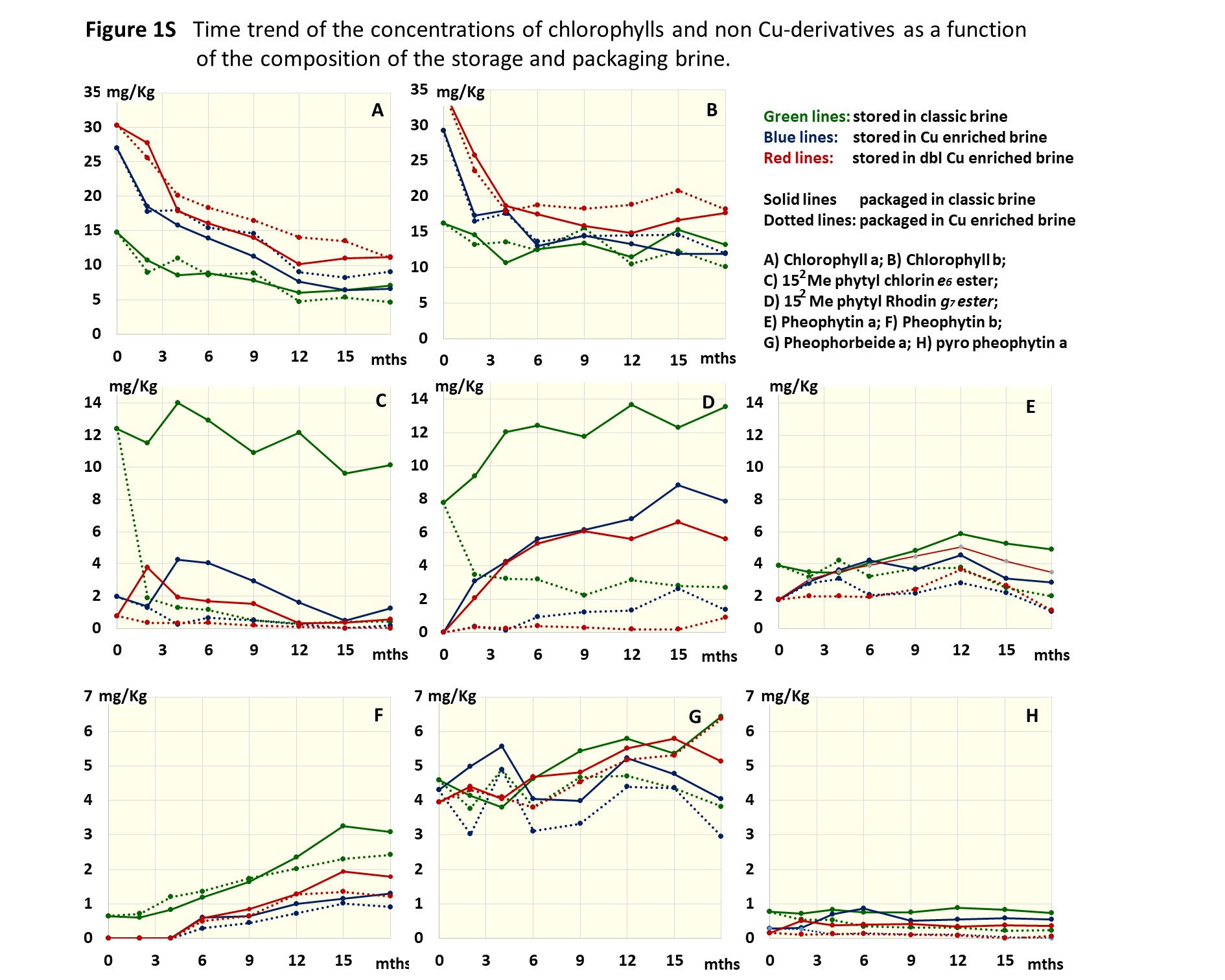

Supplement: Supplementary file 1 [file molecules-28-04250-s001.zip › Natella et al Supp Fig 1S.jpg]

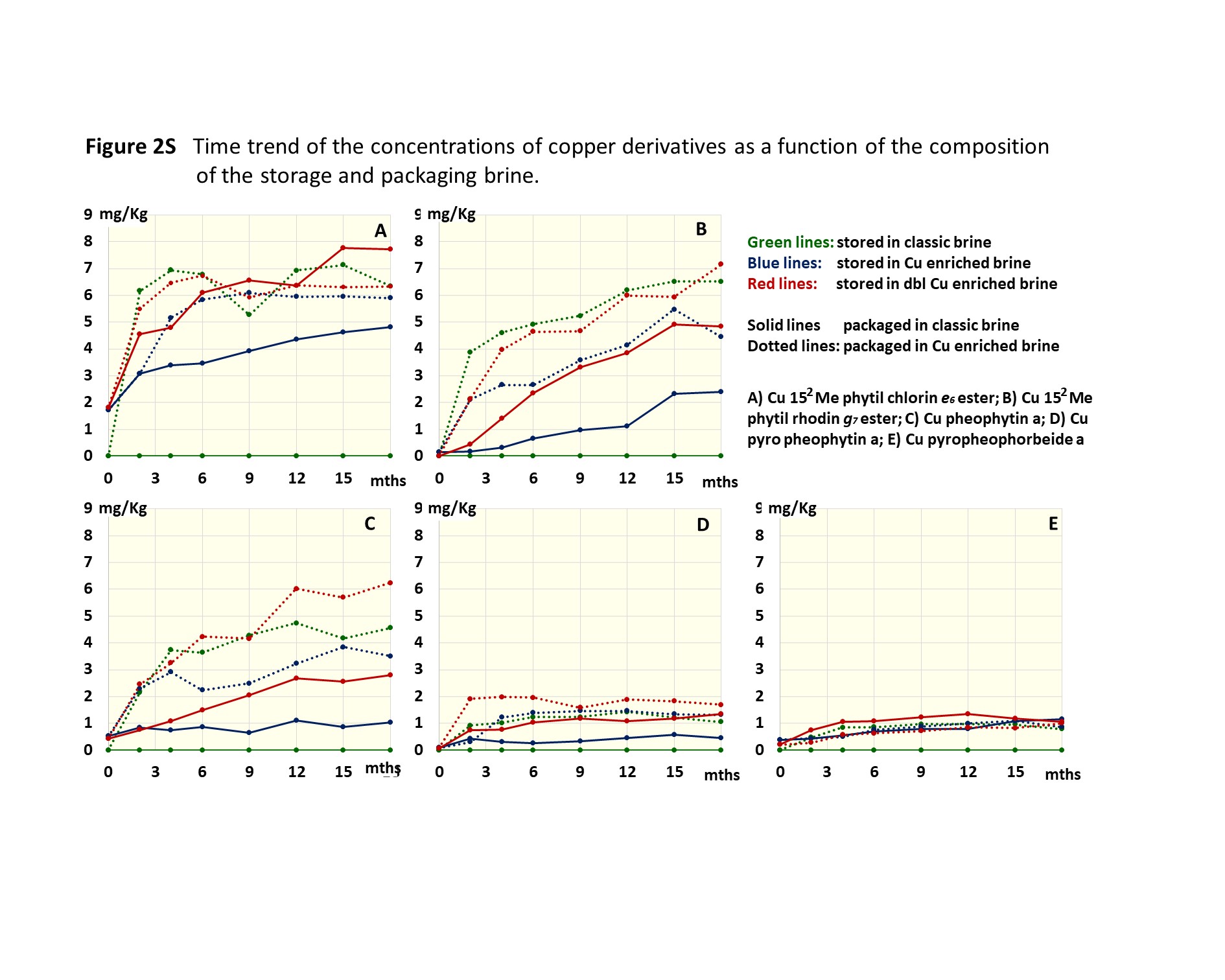

Supplement: Supplementary file 1 [file molecules-28-04250-s001.zip › Natella et al Supp Fig 2S.jpg]
